# Intestinal Microbiota in Early Life and Its Implications on Childhood Health

**DOI:** 10.1016/j.gpb.2018.10.002

**Published:** 2019-04-12

**Authors:** Lu Zhuang, Haihua Chen, Sheng Zhang, Jiahui Zhuang, Qiuping Li, Zhichun Feng

**Affiliations:** 1 Affiliated Bayi Children’s Hospital, The Seventh Medical Center of PLA General Hospital, Beijing 100700, China; 2 The First Clinical Academy of Dalian Medical University, Dalian 116011, China; 3 College of the Environment, Northeast Normal University, Changchun 130117, China; 4 National Engineering Laboratory for Birth Defects Prevention and Control of Key Technology, Beijing 100700, China; 5 Beijing Key Laboratory of Pediatric Organ Failure, Beijing 100700, China

**Keywords:** Intestinal microbiota, Immunity, Gut–brain axis, Early life, Diseases, Microbiota manipulation

## Abstract

Trillions of microbes reside in the human body and participate in multiple physiological and pathophysiological processes that affect host health throughout the life cycle. The microbiome is hallmarked by distinctive compositional and functional features across different life periods. Accumulating evidence has shown that microbes residing in the human body may play fundamental roles in infant development and the maturation of the immune system. Gut microbes are thought to be essential for the facilitation of infantile and childhood development and **immunity** by assisting in breaking down food substances to liberate nutrients, protecting against pathogens, stimulating or modulating the immune system, and exerting control over the hypothalamic–pituitary–adrenal axis. This review aims to summarize the current understanding of the colonization and development of the gut microbiota in **early life**, highlighting the recent findings regarding the role of intestinal microbes in pediatric **diseases**. Furthermore, we also discuss the microbiota-mediated therapeutics that can reconfigure bacterial communities to treat dysbiosis.

## Introduction

Trillions of microbes inhabit mucosal surfaces of the human body, and play important roles in various multiple physiological and pathophysiological processes that affect host health throughout the life cycle. Bacterial colonization, dynamic structural changes in the gut virome, as well as interactions between gut microbes and host cells are crucial steps during the developmental trajectory [1]. Furthermore, cumulative evidence has shown that microorganisms inhabiting the human intestinal tract may exert fundamental influences on infant development and the maturation of their immune system [2], [3]. These findings suggest that the risk of acquiring diseases may be programmed during the fetal developmental period and early life [4], making it imperative to explore the role of the human microbiota in early life [5], [6], [7]. For the intestinal microbial community, during the first 2.5 years of life, the abundance of Bacteroidetes continuously increases. In the meantime, number of genes associated with vitamin biosynthesis, carbohydrate utilization and xenobiotic degradation increases, together with an elevated amount of fecal short-chain fatty acids. In the end, a more stable gut microbial community structure is gradually formed [8]. It is assumed that the foundation for an adult-like intestinal microbial community is established during early life and that the early microbiota plays a dominant role in future health [9].

The following sections summarize the recently acquired evidence regarding the formation and development of the human infantile intestinal microflora, discuss the alterations in the human gut microbiome in pediatric diseases, and present strategies that could be employed to directly manipulate the gut microbiota during early life stages in humans.

## Colonization and development of the intestinal microbiota during early life

### Colonization of the intestinal microbiota before birth

**Figure 1:**
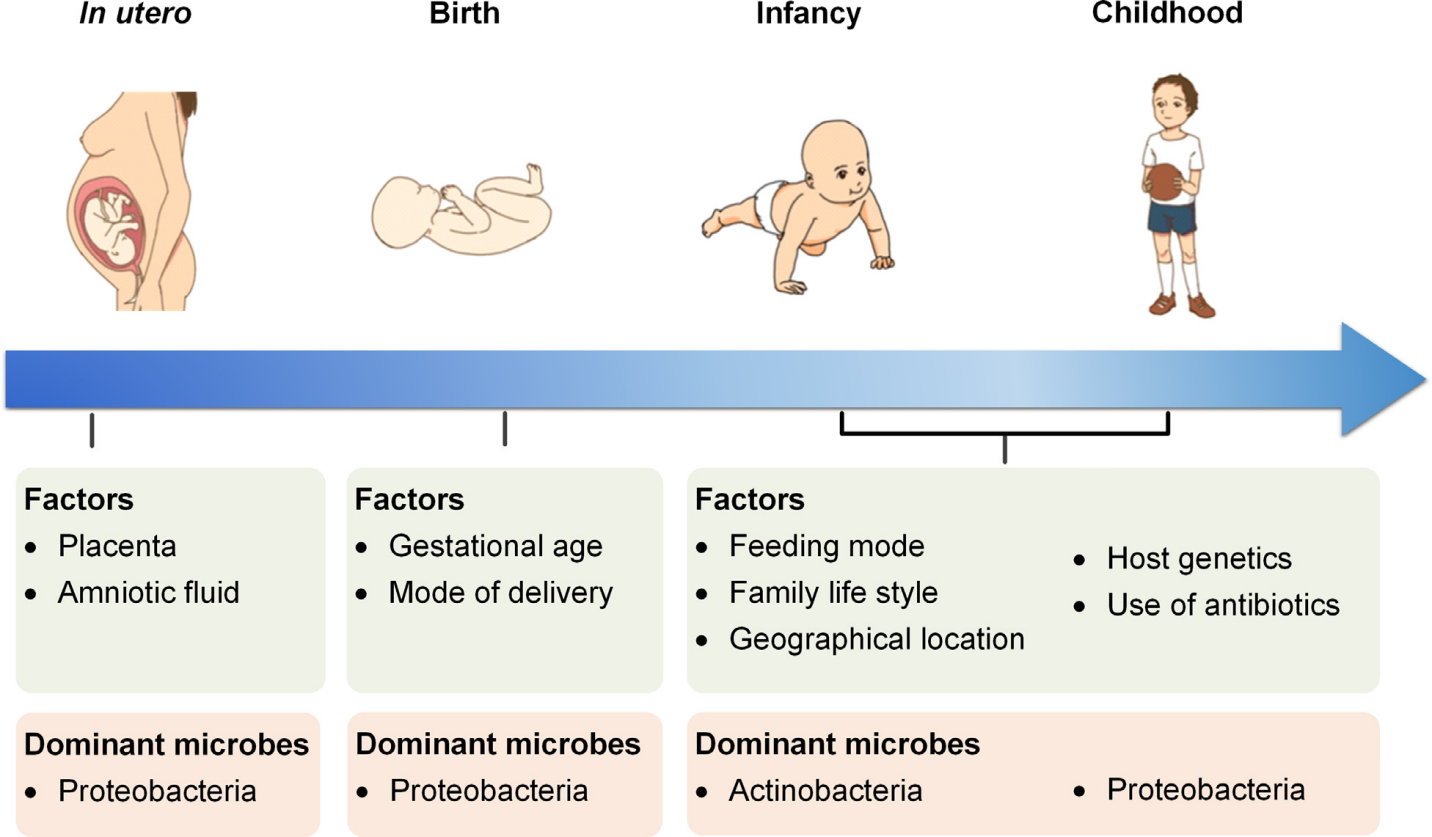
**Factors shaping the intestinal microbiota during early life and development** The presence of microbes in the placenta and amniotic fluid suggests colonization of the fetus *in utero* with a Proteobacteria-dominated microbiome. During the neonatal period, gestational age and the mode of delivery influence the microbial colonization in the newborn. Changes in age and feeding mode, family lifestyle, geographical location, genetics of the infant, as well as the use of antibiotics further configure the microbiome in early life. The microbiota becomes more diverse over time, and the dominant microbes are Actinobacteria and Proteobacteria*.*

For decades, the human fetal environment has been considered sterile under physiological conditions. Since the identification of differences in symbiotic bacteria from meconium samples between healthy normal and cesarean newborn [10], the notion that the intrauterine fetal milieu is sterile has been challenged. In 2008, researchers inoculated pregnant mice with a genetically-marked *Enterococcus faecalis* strain through the oral cavity and subsequently isolated the bacterium from the meconium of the offspring delivered by C-section [10], suggesting that maternal microbes could enter the gastrointestinal (GI) tract of the fetus. Recently, a whole-genome shotgun metagenomic study of placental specimens collected from 320 subjects under sterile conditions reveals a unique placental microbial flora that comprises nonpathogenic commensal microbes from the Tenericutes, Firmicutes, Bacteroidetes, Proteobacteria, and Fusobacteria phyla [11]. Amniotic fluid has also been reported to harbor a distinct microbial community characterized by low diversity, low richness and a predominance of Proteobacteria [12] (Figure 1). Similar to the placenta and fetus, the meconium has been previously considered sterile [13]. However, some recent studies have demonstrated that the meconium contains a complex microbiota. A study on the first meconium collected from 15 vaginally-delivered healthy term infants indicates that *Bacteroides–Prevotella* exists predominantly in the first meconium [14]. A recent microbial profiling study based on 16S rRNA high-throughput sequencing shows that regardless of the mode of delivery, the microbial population in the meconium is quite similar to that in the maternal placenta between the mother–infant pairs [15]. Despite the lack of direct evidence, these findings indicate that bacteria are transmitted to the fetus from the mother, suggesting that the manipulation of the oral and intestinal microbiota during or before pregnancy might affect not only the pregnancy outcome but also fetal and infantile health.

However, although accumulating evidence indicates that the fetus might inherit mother’s microbes even before birth, there have been controversies about the microbial colonization before birth. The “*in utero* colonization” hypothesis may need to be reconsidered since these studies were mostly conducted using molecular methods, which are not appropriate for the study of low-abundance microbial communities, due to the lack of suitable controls to evaluate contamination [16], [17].

### Development of the microbiota after birth

The neonatal and infancy periods are important stages in the establishment of the intestinal microbial community [18]. At the moment of birth, microbes colonize the newborn [19] (Figure 1). Vaginally-delivered neonates are exposed to the maternal vagina, and the fecal microbiota of these neonates is dominated by *Prevotella* spp. and *Lactobacillus* [20], [21]. Neonates born via cesarean section (CS) do not come into direct contact with the maternal vaginal microbial population and are therefore more likely to have a microbiome dominated by microbes, such as *Corynebacterium*, *Staphylococcus*, and *Propionibacterium* spp., that are derived from the maternal skin, the hospital environment, or hospital staff [21], [22], [23], [24]. During the first week after birth, a dominance of Actinobacteria (mainly comprising the genus *Bifidobacterium*) has been observed for vaginally-delivered infants, while Firmicutes has been observed as the most prevalent microbial population for CS infants [25]. Moreover, the prevalence of bifidobacteria continuously increased in both vaginallydelivered and CS infants over time [25]. In terms of food intake (Figure 1), the intestinal microbiota of neonates is significantly influenced by the feeding mode, and differences in intestinal microbes between exclusively breast-fed *vs.* formula-fed infants have been well documented [26]. As reported by several studies, the stools of breast-fed infants contain more *Lactobacilli* and bifidobacteria and fewer potential pathogens than the stools of formula-fed infants, which contain a more diverse intestinal microbial flora dominated by *Bacteroides*, *Clostridia*, *Staphylococci*, enterobacteria, Enterococci, and *Atopobium* [27], [28], [29], [30]. Oligosaccharides (such as human milk oligosaccharides, HMOs), of which human milk is a rich source, are considered to be natural prebiotics and can actively promote the growth of specific microbial species, such as bifidobacteria, in the infant intestinal microbiota [31], [32], [33], [34]. With the withdrawal of breast milk and the introduction of solid foods, the diversity of the intestinal microbiota increases, with Actinobacteria and Proteobacteria becoming the dominant components of the infant microbiota [8], [35]. In particular, the level of the dominant bifidobacteria in the microbial community decreases with the addition of solid foods [36]. This transition of the intestinal microbiota usually takes 3–5 years, with maximal shifts in the relative abundances of taxonomic groups occurring during this time [37]. The diversification of the infant microbiota progressively continues during this critical period and progresses toward an adult-like gut microbiota before becoming more complex and stable [8], [35], [38].

In addition to delivery and feeding modes, other factors, including the gestational age at birth, geographical location, family lifestyle, host genetics, and use of antibiotics, are also responsible for infant gut microbiota colonization (Figure 1). Preterm infants usually present with immature gastrointestinal, respiratory, neurological and immunological systems. Therefore, preterm infants are often exposed to drug treatments, especially the extensive use of antibiotics. These neonates usually need long-term hospitalization and receive parenteral nutrition and mechanical ventilation, which may affect the natural process of the colonization and development of the microbiota and may possibly result in a deviation in the establishment of the gut microbiota or an aberrant composition of the gut microbial flora [39]. In preterm neonates, the gut colonization of commensal anaerobic microbes is delayed. Hence, the fecal material of preterm infants contains significantly higher levels of *Enterococcus*, Enterobacteriaceae, and opportunistic pathogens than the fecal material of term neonates [25], [40], [41], [42], [43], [44].

Geographical location may affect the pattern of infant intestinal microbiota colonization [37] due to distinct cultural practices and regional diets. One study has reported the presence of a 'geographical gradient' in the intestinal microbial flora of European infants. That is, infants from Northern European countries have higher levels of bifidobacteria*,* while infants from Southern European countries have more diverse microbiota with a higher prevalence of *Bacteroides* [45].

Furthermore, evidence from one study conducted in the Netherlands reveals that the proportion of *Bifidobacterium* spp. in infants with siblings is higher than that in infants without siblings [46]. In addition, the presence of pets in the household has an impact on the composition of the gut microbiota [47].

Interestingly, a large cohort study that enrolls 1514 subjects to evaluate the impact of host genetics on the gut microbiota, pathways and gene ontology categories reveals an association between the host genotype and the taxonomy of gut microbiota in adulthood [48]. Hence, the impact of the host genotype on the colonization and development of the infant intestinal microbial flora should be considered. It is also found that the presence of a functional single nucleotide polymorphism (SNP) in the gene encoding lactase-phlorizin hydrolase (LCT) is correlated with the abundance of *Bifidobacterium*, thereby providing evidence of a gene–diet interaction in the regulation of *Bifidobacterium* abundance.

Lastly, phage predation is another powerful force that affects the structure and dynamics of the composition of the microbiota [39].

## Intestinal microbiota and pediatric diseases

### Risk of neonatal pathologies

**Figure 2:**
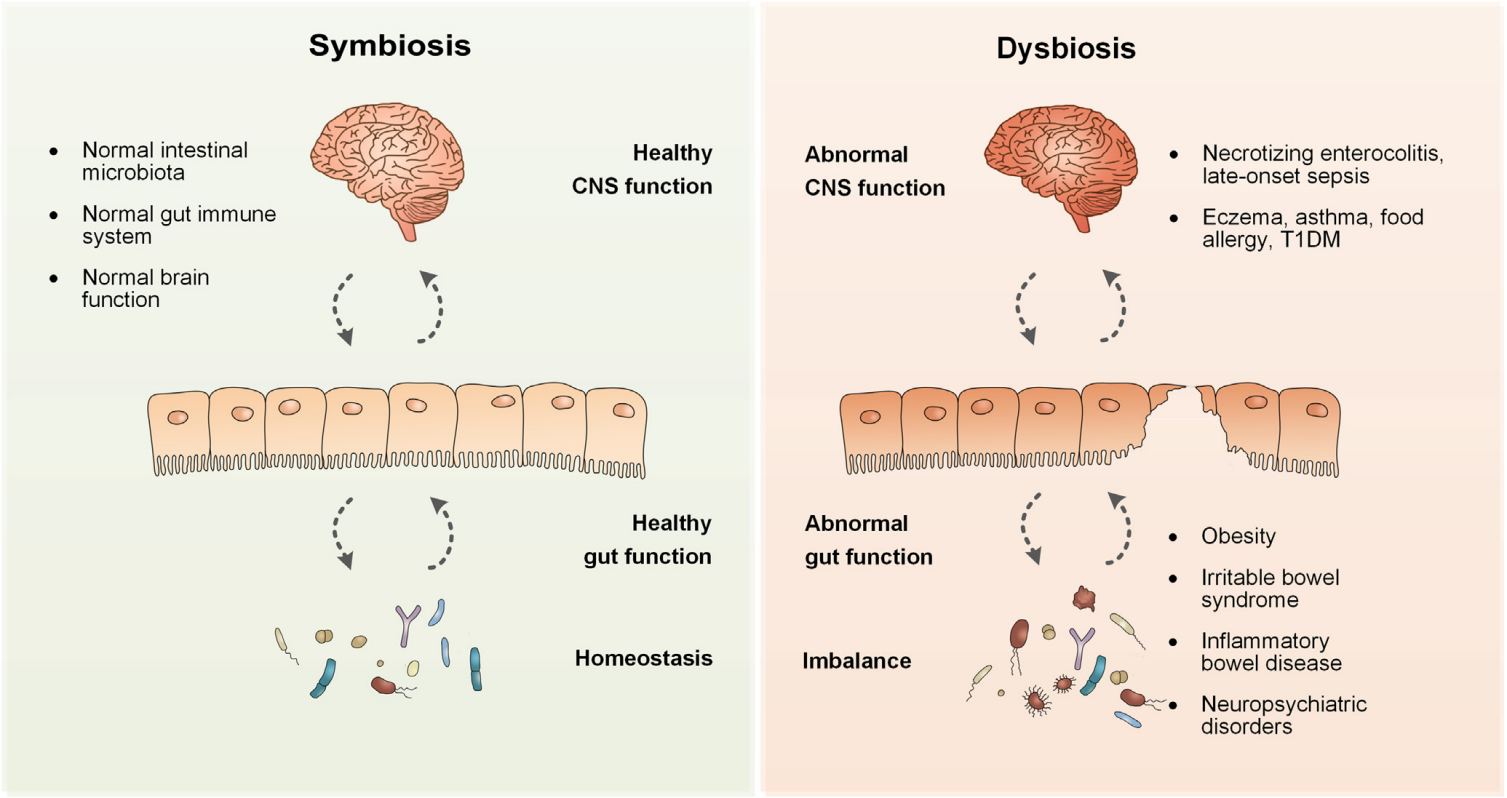
**Schematic presentation of the relationship between the gut microbiome and the brain–gut axis** The intestinal microbiota is involved in stimulating or modulating the gut immune system and exerting control over the hypothalamic–pituitary–adrenal axis indirectly. The dysbiosis of the intestinal microbiota is thought to be responsible for a series of pediatric diseases, including necrotizing enterocolitis, late-onset sepsis, eczema, asthma, food allergy, T1DM, obesity, irritable bowel syndrome, inflammatory bowel disease, and neuropsychiatric disorders. T1DM, type 1 diabetes mellitus.

Necrotizing enterocolitis (NEC) and late-onset sepsis (LOS) are two major threats to neonatal life, and their occurrence is closely associated with the intestinal microbiota (Figure 2). In premature neonates, the risk of developing NEC and sepsis is exacerbated [49], [50]. Furthermore, infants with NEC are more likely to develop LOS, mainly due to the translocation of intestinal bacterial, such as enterobacteria [42], [43]. It has been speculated that the exacerbated immune response to high-level Enterobacteriaceae may promote bacterial translocation and increase the risk of developing NEC, sepsis, and other inflammatory conditions [51]. Many metagenomic studies have shown that compared to healthy infants, infants who developed LOS harbor a less diverse microbiota and have lower levels of *Bacteroides* and *Bifidobacterium* and a predominance of enterobacteria in their intestines [52], [53], [54]. In addition, the finding that a high proportion of infants with LOS share the same bacteria in their presepsis stool provides a strong argument for bacterial translocation [55], [56]. Furthermore, some studies have shown that the diversity of the intestinal flora decreases, while the abundance of specific pathogens increases in infants who developed NEC [57], [58]. However, currently there is no consensus on this issue.

In premature infants, the microbial dysbiosis preceding NEC has been characterized by an elevated level of Proteobacteria and reduced levels of Firmicutes and Bacteroidetes [59]. Stewart et al. have reported that there is no significant difference in the mean number of total bacterial species between infants with NEC and healthy infants. However, the bacterial composition differs between these two group of infants, with *Sphingomonas* found to be predominant in infants who are diagnosed with NEC later on [60], [61]. Furthermore, the low microbial diversity may induce the overgrowth of pathogenic bacteria, which has also been considered as an important factor that contributes to the development of NEC. However, there appears to be no particular composition of intestinal microbiota that predisposes a neonate to NEC.

### Eczema

Eczema, which is the most frequent inflammatory skin condition during childhood, influences the physiological and psychological conditions of patients [62], [63]. In addition, eczema is a strong predictor of allergic disease [64], [65], [66], [67]. Improved public health reduces early exposure to microbes by reducing infections during childhood, which has been suggested to be a cause of the sustained increase in allergic diseases [68] (Figure 2). Some studies have shown that the diversity of the intestinal microbial population in infants with eczema is significantly reduced [69], [70], and the abundant pathogenic bacteria during the early stages of infancy belong to the *Enterococcus* and *Shigella* genera [71], [72]. Strikingly, high levels of *Lactobacillus* and *Bifidobacterium* are detected in infants without eczema at 1–3 months, 12 months, two years, and five years of age [71], [72], [73]. Furthermore, several species, such as *Ruminococcus gnavus* and *Faecalibacterium prausnitzii* that are known to be responsible for inflammation and atopy, are found to be markedly enriched in infants with eczema [64]. Therefore, growing evidence has shown that the gut microbiota plays an essential role in the regulation of both innate immunity and specific immunity, as well as the development of allergic diseases [73]. However, little is known about the mechanism by which the gut microbial community coevolves with the immune system in the early stages of life and how intestinal microbes affect the immune system [74].

### Asthma

Asthma, one of the most common chronic illnesses worldwide, is a complex, heterogeneous immune-mediated collection of disorders characterized by airway remodeling and chronic airway inflammation [68]. Asthma is classically associated with the hyperactivation of the T-helper 2 (Th2) arm of specific immunity. Numerous risk factors for asthma, including CS [75], antimicrobial exposure during early life [76], lack of maternal exposure to pets or livestock during pregnancy [77], formula feeding [30], [78], and maternal exposure to antibiotics during pregnancy [79], are focused around on the prenatal and early postnatal periods, suggesting that the occurrence of allergic asthma in children may be correlated with the early intestinal microbial community during the critical period of microbiological and immunological development (Figure 2). Furthermore, there appears to be a large number of immune interactions with environmental stimuli of microbial nature that affect the pathogenesis of asthma and atopy. By analyzing the feces of 298 infants between the ages of 1 and 11 months using 16S rDNA sequencing, a recent cohort study conducted in the United States shows that the dysbiosis of the neonatal intestinal microbiota might help promote CD4^+^ T cell dysfunction, which is known to be associated with childhood atopy [80]. Furthermore, another study reports that mice fed a high-fiber diet can generate a distinctive intestinal microbiota, resulting in an increased level of the short-chain fatty acid acetate. The high-fiber or acetate feeding notably suppresses allergic airway disease (AAD) by reducing the expression in mouse fetal lungs of certain genes linked to both human asthma and mouse AAD (a model for human asthma) [81]. Moreover, intestinal microbes are found to not only be associated with asthma but also reduce the diversity and community composition of the airway microbiota linked to the severity and inflammatory phenotype of asthma [82]. A further investigation of the gut and lower airway microbiota in individuals with asthma may help develop more effective ways to prevent and treat asthma.

### Food allergy

The incidence of food allergy has been increasing in recent decades, with an estimated prevalence in the developed world of approximately 10% [83]. In particular, the prevalence of IgE-mediated food allergy has increased sharply, especially in infants and young children [84]. The cause of this increase remains unknown. However, one of the possible factors is the microbiota that colonizes the human gut during early infancy (Figure 2). Recent studies have shown that there is an aberrance of intestinal microbial flora in infants and children with food allergy. A reduced richness of the intestinal microbiota and an increased level of *Bacteroides* species are observed in patients with tree nut or peanut allergy, when compared to nonallergic control subjects [85]. A longitudinal study investigates the intestinal microbiome composition in infants aged 3–6 months and indicates that early infancy is an important period during which the intestinal microbiota may shape the outcomes of food allergy in childhood. This study reveals a difference in the intestinal microbiome composition between subjects who resolve their milk allergy by eight years old and those whose milk allergy persists. In particular, a higher enrichment of Firmicutes and Clostridia is found in the former group of infants than the latter group [86]. Animal experiments have also confirmed such alterations in the composition of the microbiota. Ovalbumin (OVA)-sensitized food allergy-prone mice (Il4raF709) harbor a particular gut microbiota signature characterized by synergistic alterations in the composition of several bacterial families, including Porphyromonadaceae, Lactobacillaceae, Rikenellaceae, and Lachnospiraceae [87]. When the gut microbiota is transferred from food allergy-prone mice (with a gain-of-function mutation in the IL-4 receptor α chain) to wild-type germ-free mice, the germ-free mice display an exaggerated anaphylactic response to food allergens [87]. In addition, accumulating evidence has shown that the structure and metabolic activity of intestinal microbes are strongly correlated with the processes involved in allergic diseases and the protective tolerogenic pathways [88], [89].

### Type 1 diabetes mellitus

Type 1 diabetes mellitus (T1DM), also called diabetes mellitus type I (DM1), is an immune-mediated metabolic disease characterized by the progressive destruction of pancreatic islet β cells. The mode of delivery [90], [91], diet in early life [92], [93], and antibiotic usage [94] have been indicated to be responsible for the increased incidence of DM1, and ample evidence has supported the proposed role of microbes, which are influenced by all of the key environmental factors involved in the development of DM1 (Figure 2). Compared to healthy controls, DM1 patients harbor a less diverse and less stable intestinal microbiota [95], [96], and changes in the proportion of Firmicutes to Bacteroidetes are observed in DM1 patients [95], [96], [97], [98]. It has also been found that prediabetic children harbor more Bacteroidetes than controls [97]. Experiments in rodents have shown that continuous low-dose antibiotics or pulsed therapeutic antibiotics in early life alters the intestinal microbial flora and T cell populations, increases the risk of DM1, and facilitates the occurrence of DM1 in a nonobese diabetic murine model [99]. Furthermore, according to the present knowledge, intestinal microbiota could affect the risk for T1D in a two-step process. Initially, the intestinal tract is colonized by a microbial population that could not sufficiently promote the development of the immune system. Then, the intestinal microbial diversity is reduced, the subsequent dysbiosis predisposes the young child to T1D, and the inadequately functioning immune system results in progressive β cell autoimmunity, eventually leading to the occurrence of clinical T1D [100]. Hence, more mechanistic studies are required to establish the causal relationship between the microbiota and DM1.

### Obesity

Obesity does not only influence the occurence but also the development of metabolic diseases, such as cardiovascular diseases, diabetes mellitus type II (DM2), cancers, and osteoarthritis [101]. Recent studies involving human subjects and animal models have demonstrated that dysbiosis of the intestinal microbiota is involved in the development of obesity (Figure 2). The number of bifidobacteria in fecal samples during infancy is higher in normal-weight children, while the amount of *Staphylococcus aureus* is higher in overweight children [102]. In addition, a cohort study enrolling 909 one-month-old children who have been followed up from one month old to 10 years of age reveals that the colonization level of *Bacteroides fragilis* at one month of age is associated with elevated body mass index (BMI) z-scores in children up to 10 years of age [103]. Experimental models have suggested several mechanisms underlying the correlations of intestinal microbes with obesity and other metabolic diseases. These include increased dietary energy acquisition, promotion of fat deposition, locomotor activity modification, satiety effects, and systemic inflammation activation [104], [105], [106], [107]. The role of the microbiota in the genesis of obesity may widen the views of weight control and obesity treatment.

### Irritable bowel syndrome

Irritable bowel syndrome (IBS) is one of the most common GI diseases in industrialized countries, with a prevalence of approximately 10%–15% [108]. Although the etiology of IBS has not been completely understood, it is generally deemed a multifactorial disease that develops through the interaction of the host with environmental factors. The risk of IBS increases with the occurrence of gastroenteritis [109]. In addition, the association with prior antibiotic use [110] supports the importance of gut microbes in the development of IBS (Figure 2). Accumulating evidence has shown that alterations occur in the intestinal microbiota of IBS patients in comparison with the healthy controls [111], [112], [113], [114], [115]. Recently, the Rome Team Working Group creates a clinical guideline about the manipulation of the gut microbiota in IBS [116], concluding that there is sufficient evidence to support the view that the gut microflora is disturbed in IBS patients. Despite the growing consensus on the association between IBS and the gut microbial community, there is a lack of general consensus among current studies, and the specific microbial influence on IBS remains elusive. Some clinical trials have suggested that changes in specific microbe populations in early life are, to some extent, associated with the development of gastroenterological inflammation in senior life [117], [118], [119], [120]. However, since there is no convincing evidence for the chronic effect of dysbiosis during early life on the incidence of IBS in children and adults, causality could not yet be established [39]. Future human epidemiology studies and experimental studies with animal models are required to gain deeper insights into the microbiota-mediated physiological and immune responses that may be associated with the development of the disease.

### Inflammatory bowel disease

Inflammatory bowel disease (IBD) is a chronic complex disorder in children and adults that comprises two major immune-mediated disorders: Crohn’s disease (CD) and ulcerative colitis (UC). IBD requires a range of medications and surgical techniques to treat the symptoms, promote growth and development, and support a restriction-free life. However, the burden of IBD in children can be high. Although the pathogenesis of IBD remains unclear, some recent studies have suggested that the individual genetic susceptibility, external environment, immune responses, and gut microbial flora are all involved and functionally integrated in the genesis and development of IBD [121], [122], [123]. IBD has been reported to be attributable to an altered gut microbiota composition, as represented by the loss of members belonging to the Bacteroidetes and Firmicutes phyla and an increase in Proteobacteria [124]. By quantitatively analyzing the solitary and synergistic effects of dietary macronutrients and microorganisms on the development of colitis, a recent study finds that the concentration of fiber and protein has the greatest impact on the development of colitis [125]. Host–microbial interactions, specifically those related to the IL-23/Th17 pathway, T cell activation, and microbial recognition and autophagy, are also considered to be an important component of the pathogenesis of IBD [126], [127], [128]. Currently, it remains unclear how the complex interactions between intestinal microbes and diet affect the development of IBD [125]. More convincing and consistent data are required to better understand the chronic impact of early-life dysbiosis on the development of IBD and the causal relationships among the microbiota, diet, and IBD [39].

### Neuropsychiatric disorders

Neuropsychiatric disorders are a major threat to global health and are considered to be multifactorial disorders that are caused by specific environmental factors in individuals with genetic susceptibility. Indeed, studies on the underlying mechanisms of neuropsychiatric disorders have demonstrated that the intestinal microbiota could affect brain physiology, which in turn affects behavior through the humoral and neural pathways of gut–brain communication, suggesting that the gut microbial community is an important node in the system [129] (Figure 2). In addition, several other studies have also suggested that the activity of the intestinal microbiota could alter the host epigenome and thereby impact the gene expression associated with neuronal plasticity, learning, memory, and neurogenesis, as well as disorders such as schizophrenia, cognitive dysfunction, and depression [130], [131]. Studies in recent years have shown that the intestinal microbiota is critical in maintaining the healthy functional state of microglia, which is essential for the prevention of neurodevelopmental and neurodegenerative diseases [132], [133]. Studies have shown that germ-free mice exhibit deficiencies in social behavior and that the gut microbial population is critical in the development of neuronal circuits underlying motor control, social responses, and anxiety behaviors [134], [135]. Furthermore, recent studies have indicated that the intestinal microbiome of children with autism exhibits different levels of Firmicutes and Bacteroidetes [136], [137]. In addition, schizophrenia and attention deficit hyperactivity disorder have also been considered to be associated with intestinal microbes [138], [139]. To clarify the underlying pathogenesis, further work is needed to elucidate the alterations in the intestinal microbiome and the complex gene–environment interactions involved in the occurrence of different neuropsychiatric disorders. It is noteworthy that the intestinal microbial community is possibly more “medically” accessible and modifiable than the human genome, which may provide a promising strategy for the prevention and/or treatment of neuropsychiatric diseases [140].

## Manipulation of intestinal microbes

### Antibiotics

Antibiotics, which are usually used to prevent or treat infections that are not necessarily caused by a specific pathogen, can efficiently deplete the intestinal microbiota. Neonates with NEC have a high risk of infection caused by microbes of the intestine, and antibiotics are often used to prevent or treat these infections [141]. For the treatment of pediatric IBD, administration of single antibiotic is beneficial to patients with complications, such as fistulae and abscesses, whereas broad antibiotic combinations might improve clinical outcomes [142], [143]. However, there is a great risk associated with the use of antibiotics in young children. Ample evidence has shown that antibiotics affect our ability to resist infection, function of the immune system, and our capacity to process food [144]. The disruption of the intestinal microbiota may result in long-term health consequences, including reduced production of vitamins, decreased absorption of nutrients, and increased risks of diabetes, asthma, obesity, and infections [145].

### Prebiotics and probiotics

Orally supplied prebiotics and probiotics are the most common ways to influence intestinal microbiota development in early life stages. Prebiotics are defined as compounds that result in the ‘*selective stimulation of growth and activity of one or more microbial genus or species in the gut microbiota that confer health benefits to the host*’ [146], while probiotics are defined as ‘*living microorganisms that confer a health effect on the host when they are consumed in adequate amounts*’ [147]. The currently available prebiotics include human milk oligosaccharides, inulin, fructo-oligosaccharides, and galacto-oligosaccharides [148]. The currently available probiotics include *Bifidobacterium* spp. and *Lactobacillus* spp. [148]. By modulating the intestinal microbiota, prebiotics confer a health effect on the host. Probiotics strengthen the gut epithelial barrier via competitive adherence to the mucosa and epithelium, the mucosal IgA response, the secretion of antimicrobial substances, the downregulation of proinflammatory pathways, the generation of anti-inflammatory cytokines, and the modulation of the immune system [29], [147], [149], [150]. Recent studies have suggested that probiotics could be protective against the progression of pediatric diseases and disorders, including allergies, GI infections, obesity, and even upper respiratory infections [149], [151], [152], [153], [154], [155], [156], [157], [158], [159]. Interventional studies further demonstrate that probiotics can mitigate the severity of certain diseases, but the optimal intervention for each disease remains poorly understood [160], [161]. Although probiotics can alleviate allergic symptoms in some cases, they are generally not efficacious in modulating the composition of the intestinal microbes [162]. There is evidence that the combination of *Streptococcus thermophiles* and bifidobacteria is effective to prevent antibiotic-associated diarrhea in children [163]. Furthermore, synthetic biology allows the engineering of probiotics and commensal microbes to possess novel therapeutic functions. For instance, expression of the fusion protein HSP65-6P277 reduces DM1 onset in nonobese diabetic mice [164], and the oral administration of recombinant *Lactococcus lactis* that is engineered to express HSP65-6P277 in non-obese diabetic mice could improve glucose tolerance and markedly reduce insulitis [165]. Although the applications of prebiotics and probiotics remain promising, the timing of their administration, the effect of different strains and combinations of strains, engineering, safety, and the determination of whether these probiotics would be more efficacious in combination with prebiotics should be addressed in future studies.

### Dietary modification

Nutrients can exert short-term and long-term effects on colonization patterns of the intestinal microbes in infants by shaping the composition of the microbial flora [20], [166], [167]. Mounting evidence has shown that ingested dietary components are associated with the development of IBD, DM2, and atherosclerosis [168]. The greatest change in intestinal microflora occurs with the introduction of solid foods, indicating that diet should be considered a central determinant of the gut microbiota [8], [169], [170]. Interestingly, there has long been a concept of “drug and food homology” in the traditional Chinese medicine. One aspect of the concept is that food is a type of medicine and that a proper diet or certain foods can maintain the balance and health of the body and prevent or mitigate the development of some diseases [171]. In modern medicine, dietary modification is increasingly recognized as a relatively simple method of modifying systemic inflammation by changing the gut microbiota [74], [168]. Exclusive enteral nutrition (EEN) is a dietary therapy that has been used as a first-line therapy for pediatric CD by replacing normal dietary components with a formula exclusively composed of liquid nutrients, with the intent of normalizing inflammatory markers and inducing clinical remission [172]. In addition, some recent studies have shown that dietary fibers possess well-documented anti-inflammatory properties, which can partially explain the fiber-induced effects on the gut microbial flora [173], [174], [175], [176]. Several studies using preclinical models has demonstrated how fermentable fiber supplementation can modify disease outcomes via microbiota-induced changes in the production of specific anti-inflammatory metabolites [177], [178]. Nevertheless, more studies are urgently needed to increase our understanding on how different diets shape the microbiota and modify health outcomes.

### Fecal microbiota transplantation

Fecal microbiota transplantation (FMT) is defined as the transfusion of a fecal suspension from a healthy donor into the GI tract of a recipient patient to restore the normal diversity and function of the intestinal microbiota. The fecal microbiota can be placed in patients by colonoscopy [179], [180], nasogastric or nasoduodenal tubes [181], enemas [182], or oral capsules [183], [184]. Due to the technical advances in the metagenomic sequencing of the intestinal microbiota and the growing understanding of its composition and function, FMT has attracted increasing interest and attention in recent years. Although FMT remains poorly understood, it is no longer considered an “alternative” and last-resort of medical practice and is now gaining mainstream acceptance as a valuable therapy with biological plausibility [185]. Furthermore, this therapy has been proven to be able to reconstitute a normally functioning microbial community [162], [181], [186], [187]. By providing the patient with a balanced microbiota from a suitable donor, FMT corrects the imbalanced gut microbial flora that plays an important role in the pathogenesis of *Clostridium difficile* infection (CDI). Across a series of studies on recurrent CDI, symptom resolution has been observed in 85% of patients receiving FMT [188]. Moreover, considering the interactions between the gut–brain axis and intestinal microbes, FMT has been regarded as a possible therapy for some mental illnesses, such as autism spectrum disorder [189]. However, the microbial composition for FMT has not been perfectly defined. Therefore, the microbial structure or functional profile related to improved clinical outcome needs to be clarified to identify the preferred assemblages. Thus, future studies should focus on determining the range of “healthy” microbial flora and the development of the criteria for assessing the optimal composition.

## Perspectives

A balanced symbiosis of the gut microbiota is closely associated with human health, although, this large, diverse, and dynamic population has long been neglected. With the improvement in observational and investigative techniques, it has been recognized that symbiotic microbial communities interact with most host organs and have unique compositions and functional characteristics at different life stages. The gut microbiota plays an important role in the maturation of the immune system, especially in the early stages of life, and during infant growth and development.

It has been assumed that the process of colonization and development of gut microbiota in early life is linked to diseases in later life. Accumulating evidence from different studies has shown that the occurrence of a disease is often preceded by early alterations of the microbiota. Given the potential individual-specific physiological role, the microbiome is suggested to be a good predictor of disease risk. We envision that the risk of a number of complex lifestyle-related and age-related human disorders, such as metabolic, inflammatory, and neurodegenerative diseases, could be predicted and stratified by the characterization of a disrupted microbiota. The nature and mechanisms by which the microbiota changes during life and the means by which those changes affect biological pathways should be better understood.

## Competing interests

The authors have declared no competing interests.
